# A retrospective study on predicting clinically significant prostate cancer via a bi-parametric ultrasound-based deep learning radiomics model

**DOI:** 10.3389/fonc.2025.1538854

**Published:** 2025-04-08

**Authors:** Xiang Liu, Zhong-Xin Zhang, Bing Zheng, Min Xu, Xin-Yu Cao, Hai-Ming Huang

**Affiliations:** ^1^ Department of Ultrasound, The Second Affiliated Hospital of Nantong University, Nantong, Jiangsu, China; ^2^ Department of Urology Surgery, The Second Affiliated Hospital of Nantong University, Nantong, Jiangsu, China

**Keywords:** bi-parametric, ultrasound, deep learning, radiomics, prostate cancer

## Abstract

**Purpose:**

This study aimed to establish and evaluate a model utilizing bi-parametric ultrasound-based deep learning radiomics (DLR) in conjunction with clinical factors to anticipate clinically significant prostate cancer (csPCa).

**Methods:**

We retrospectively analyzed 232 participants from our institution who underwent both B-mode ultrasound and shear wave elastography (SWE) prior to prostate biopsy between June 2022 and December 2023. A random allocation placed the participants into training and test cohorts with a 7:3 distribution. We developed a nomogram that integrates DLR with clinical factors within the training cohort, which was subsequently validated using the test cohort. The diagnostic performance and clinical applicability were evaluated with receiver operating characteristic (ROC) curve analysis and decision curve analysis.

**Results:**

In our study, the bi-parametric ultrasound-based DLR model demonstrated an area under the curve (AUC) of 0.80 (95%CI: 0.70-0.91) in the test set, surpassing the performance of both the radiomics and deep learning models individually. By integrating clinical factors, a composite model, presented as the nomogram, was developed and exhibited superior diagnostic performance, achieving an AUC of 0.87 (95%CI: 0.77-0.95) in the test set. The performance exceeded that of the DLR (*P* = 0.049) and the clinical model (AUC = 0.79, 95%CI: 0.69-0.86, *P* = 0.041). Furthermore, the decision curve analysis indicated that the composite model provided a greater net benefit across a various high-risk threshold than the DLR or the clinical model alone.

**Conclusion:**

To our knowledge, this is the first proposal of a nomogram integrating ultrasound-based DLR with clinical indicators for predicting csPCa. This nomogram can improve the accuracy of csPCa prediction and may help physicians make more confident decisions regarding interventions, particularly in settings where MRI is unavailable.

## 1 Introduction

Prostate cancer (PCa) has become a common malignant tumor in men globally, with its incidence and mortality rates increasing each year (1). As the global population ages and medical technology advances, the need for effective screening and early diagnosis has become more critical. Nonetheless, existing diagnostic techniques like prostate-specific antigen (PSA) testing and digital rectal examination (DRE) have shortcomings in precisely detecting clinically significant prostate cancer (csPCa) (2). These traditional means often lead to misdiagnosis, underdiagnosis, and over-treatment, imposing unnecessary psychological and physical burdens on patients.

In the past few years, multiparametric magnetic resonance imaging (mpMRI) has turned into a popular method for PCa screening and diagnosis. By integrating various imaging modalities, mpMRI significantly improves the detection and grading of PCa, particularly in lesion localization and preoperative assessment (3). However, the high cost, complex equipment requirements, and limited sensitivity in detecting small-volume or low-grade tumors restrict its broader clinical application (4).

Multiparametric ultrasound (mpUS), a cost-effective and easy-to-operate imaging modality, has garnered increasing attention as an alternative. By combining imaging techniques like grayscale ultrasound, elastography, and contrast-enhanced ultrasound, mpUS provides structural, stiffness, and hemodynamic information about prostate tissue, offering additional diagnostic value in the early detection of PCa (5). In a prospective, multicenter study, Grey et al. analyzed 257 patients suspected of PCa and compared the diagnostic accuracy of mpUS with mpMRI (6). They found that the diagnostic rate of csPCa with mpUS was only 4.3% lower than that of mpMRI, with diagnostic rates of 26% for mpUS and 30% for mpMRI. Combining both imaging methods further increased the diagnostic rate to 32%.

The advent of radiomics and deep learning technologies has introduced new opportunities for improving csPCa diagnosis (7, 8). Radiomics combines medical imaging, computer science, and statistics to extract quantitative features from images, revealing subtle patterns not easily detected through traditional visual analysis (9). Deep learning algorithms, capable of automatically identifying complex imaging features, further enhance the accuracy in diagnosing csPCa (10).

Given the recent developments in these approaches, we performed a retrospective study to establish and evaluate a bi-parametric ultrasound-based deep learning radiomics (DLR) aimed at improving the accuracy of csPCa diagnosis. This model could provide an alternative diagnostic tool for physicians, especially in settings where MRI is not available.

## 2 Materials and methods

### 2.1 Patient demographics

**Figure 1:**
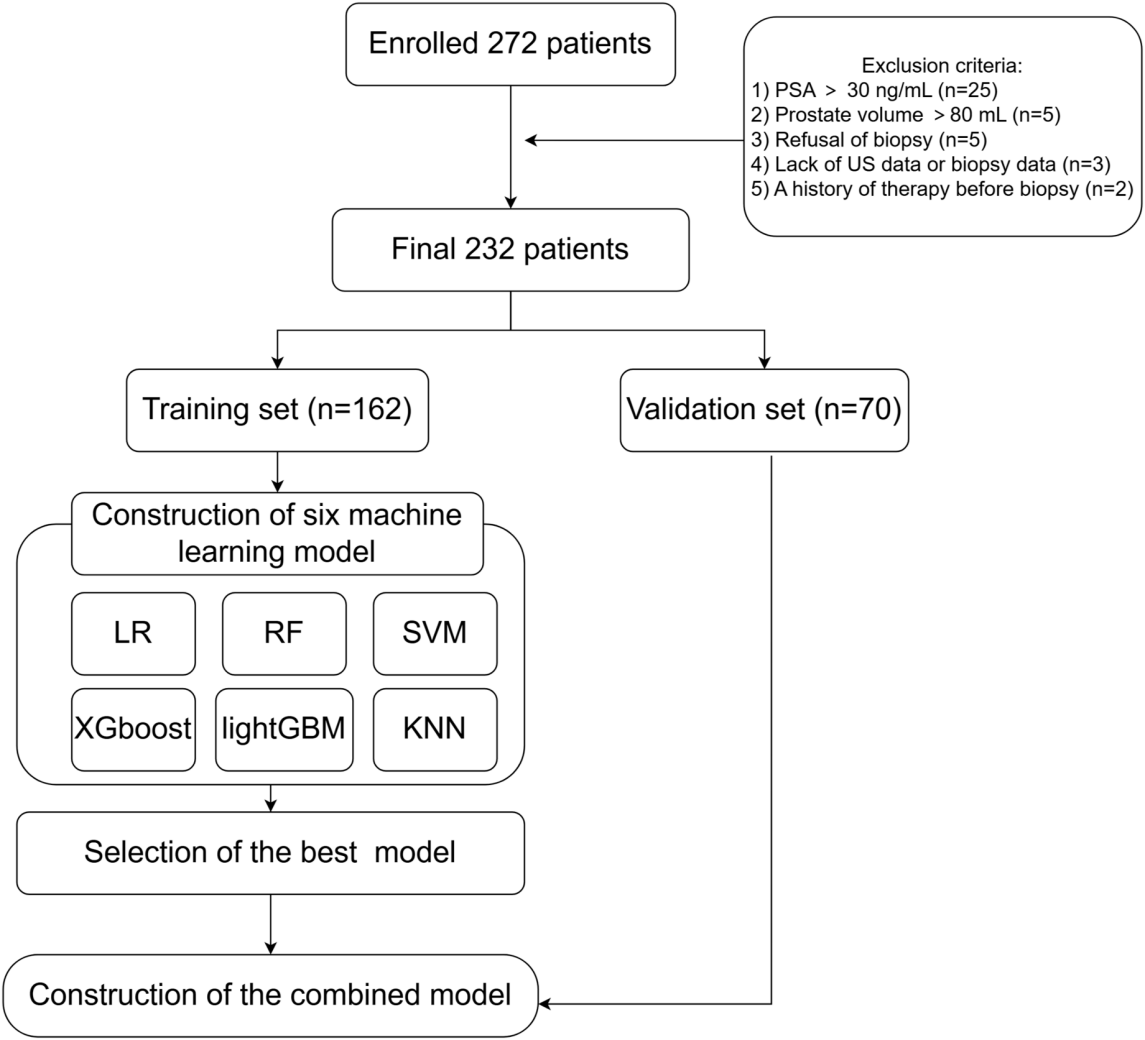
Flow diagram of the study population.

From June 2022 to December 2023, 272 patients suspected of having PCa, due to PSA rise and/or positive DRE, were retrospectively enrolled at the Department of Urology, Second Affiliated Hospital of Nantong University. Below are the criteria for inclusion: 1) elevated PSA; 2) received bi-parametric ultrasound including both grey scale and shear wave elastography (SWE) followed by prostate biopsy. Below are the criteria for exclusion: 1) PSA > 30 ng/mL; 2) absence of bi-parametric ultrasound and/or biopsy data; 3) history of radiotherapy or endocrine therapy prior to biopsy; 4) prostate volume > 80 mL. Finally, participants were randomly allocated to the training and test cohorts with a 7:3 distribution. The flowchart is shown in **Figure 1**.

### 2.2 Ethics

This research received approval from the Ethics Committee of the Second Affiliated Hospital of Nantong University (2022KT100) and was performed in line with the ethical standards set by the 1964 Declaration of Helsinki.

### 2.3 Transrectal bi-parametric ultrasound examination

The apex, middle, and base of the prostate were examined using B-mode ultrasound and SWE for each patient. Two seasoned radiologists performed the examination with an Aixplorer^®^ Ultrasound scanner (Supersonic Imagine, Aixplorer V, France).

Following the measurements of prostate volume (volume = length × width × height × 0.52), transverse and sagittal scans of the entire prostate were recorded by B-mode ultrasound. The presence of calcifications, cysts, and hypoechoic lesions indicated abnormal echo patterns. The operator visually identified and saved images of the prostate’s apical, middle, and base transverse planes. If any prostate areas appeared more suspicious than these selected planes, they were captured and stored.

Before SWE imaging, settings for maximum penetration and optimal elasticity were adjusted as needed. The SWE box scanned each predefined transverse plane on one side or both sides for full prostate coverage. A stable signal was maintained with the sensor held steady for 5 seconds. If areas outside the planned imaging plane appeared suspicious, they were also examined.

### 2.4 Biopsy procedure and pathology

A radiologist with ten years of experience performed TRUS-guided trans-perineal prostate biopsies using a Mylab Twice Ultrasound scanner with a 5.5-10 MHz probe. Local anesthesia was administered with 10 mL of lidocaine via a 22 G needle. An 18-G biopsy gun from Bard, capable of penetrating 22 mm, was used.

The “12+X” biopsy, consisting of a 12-core systematic biopsy and targeted biopsies for suspicious regions detected by SWE or TRUS, was performed for each individual. A systematic biopsy was performed in accordance with predefined transverse planes, utilizing visual estimation by an experienced radiologist. This procedure involved the insertion of a needle into 12 designated regions of the prostate (11). In addition to the previously mentioned 12 needles, three or four additional needles were inserted into the area of suspicion.

Pathologists, unaware of clinical and ultrasound results, evaluated biopsy samples. A Gleason score (GS) was recorded upon confirming PCa, with a score of 3 + 4 or higher indicating clinical significance (12).

### 2.5 Regions of interest segmentation

**Figure 2:**
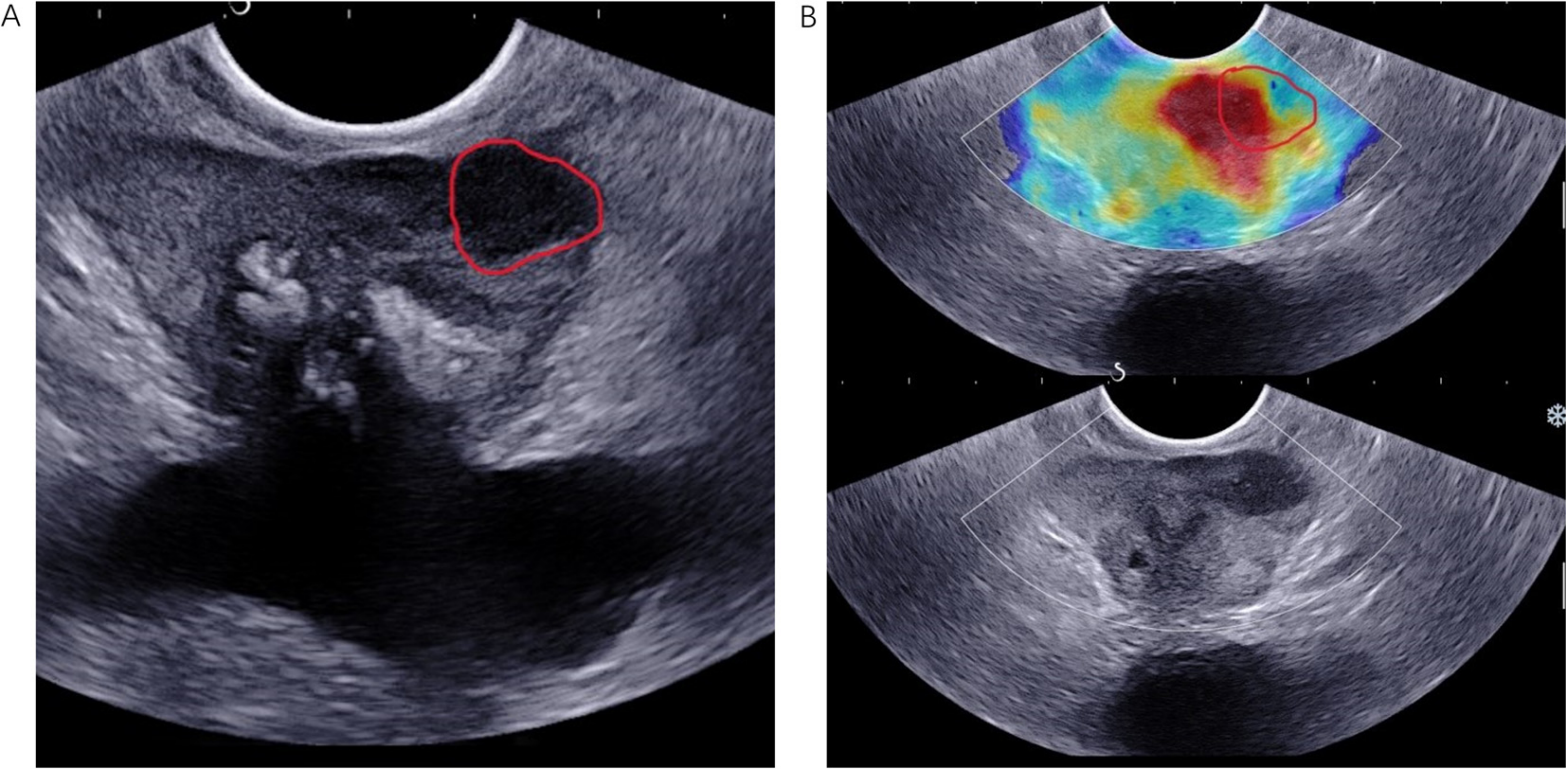
TRUS B-mode imaging **(A)** and SWE imaging **(B)** were conducted from the same anatomical location in an 83-year-old patient diagnosed with PCa, characterized by a fPSA level of 0.78 ng/mL, a tPSA level of 13.7 ng/mL, and a biopsy Gleason score of 4 + 3 = 7. The ROI, delineated by a red solid line, was identified in both the B-mode ultrasound and SWE images. PCa, prostate cancer.

To maintain data consistency and comparability, the ultrasonic images were standardized prior to segmentation. The boundary of prostate lesion was manually drawn as region of interest (ROI) using 3D Slicer software (version 5.7.0, 3D Slicer image computing platform | 3D Slicer). For consistency in the ROIs of bi-parametric ultrasound images, the identical standards were rigorously performed, and the same expert visually checked them. The method of detail segmentation referred to the study by Liang et al. (13), and determining the location and size of the lesion is roughly as followed: 1) using detailed prostate biopsy records (puncture site and depth) and pathology findings to identify the lesion’s location and nature; 2) matching pathology descriptions with TRUS images; 3) applying B-mode ultrasound ROIs to SWE images due to unclear tumor boundaries in SWE; 4) In cases of csPCa, SWE was utilized to identify ROIs corresponding to suspicious areas, particularly when B-mode imaging failed to reveal these areas. A key point in ROI labeling for multifocal PCa is using pathology results to identify the lesion with the highest GS value; if GS values are identical, the largest lesion was selected. **Figure 2** illustrated lesion segmentation for enrolled patients. Also, specialized personnel made sure that the segmentation and related pathological results were accurate.

### 2.6 Feature extraction of radiomics and deep learning features

The PyRadiomics package (version 2.1.2) was employed to extract radiomic signatures, encompassing both original and wavelet-transformed features. The study was designed in accordance with the Image Biomarker Standardization Initiative (IBSI) reporting guidelines (14). Extracted radiomics features consisted of First Order Features, Shape-Based Features, and Texture-Based Features.

ResNet-50, pre-trained on the extensive and annotated ImageNet database, was chosen as the base model for feature extraction. The network’s final fully connected layer was taken out, and the average pooling layer was applied to extract maximum values from each feature map layer, thus converting them into raw values.

Intraclass correlation coefficient (ICCs) was employed to appraise the consistency of lesion segmentation between and within observers. A cohort of 50 patients was randomly selected to assess inter-observer consistency, while an additional segmentation was conducted by a radiologist one week later to evaluate intra-observer consistency. Two radiologists, each having extensive experience in diagnosing prostate ultrasounds, delineated the ROIs.

### 2.7 Feature selection of radiomics and deep learning features

Feature scaling was performed utilizing the z-score method, which transformed the feature data within the training set into a distribution characterized by a mean of 0 with a standard deviation of 1. The goal of the program was to identify the most significant features associated with csPCa using 1702 radiomics features and 1024 deep learning features. The feature selection process involved several statistical and machine learning techniques to ensure robustness and reduce dimensionality. Initially, the Mann–Whitney U test was performed to identify features significantly linked to the outcome, which set a common p-value threshold of 0.05, ensuring conservative selection and maintaining their statistical reliability within imaging data. Next, Spearman’s rank correlation detected highly correlated features. If the coefficient between any two features exceeded 0.9, one of them was excluded from the analysis. Only the features that were significantly associated with the outcome and had a p-value below the threshold were retained. Then, the Lasso regression with 10-fold cross-validation was employed to remove features with zero-weight. The final feature selection was based on the lambda.1se criterion, which helps simplify the model by balancing predictive performance and complexity. Finally, feature permutation importance was assessed using a random forest to identify valuable features. A stepwise feature selection approach was applied, progressively expanding the feature subset while evaluating the area under the curve (AUC) to determine the optimal combination (**Supplementary Figure S1**). This process aimed to maintain classification performance while reducing the number of features, preventing overfitting, and enhancing the model’s generalization ability.

### 2.8 Model construction

Six models based on deep learning radiomics (DLR)—specifically, Support Vector Machine (SVM), Light Gradient Boosting Machine (LightGBM), Extreme Gradient Boosting (XGBoost), Random Forest (RF), K-Nearest Neighbors (KNN), and Logistic Regression (LR)—were developed for both the training and test sets to identify the model with the optimal AUC. Ultimately, LR was chosen due to its superior AUC performance on the test set. The weight coefficients of the selected features were determined through multivariate analysis, leading to the derivation of a formula to compute the radiomics score, deep learning score, and DLR score. Univariate and multivariate analyses were employed to identify independent clinical risk factors. An integrated model, incorporating the clinical factors and DLR score, was constructed using LR and is represented as a nomogram.

### 2.9 Statistical analysis

**Figure 3:**
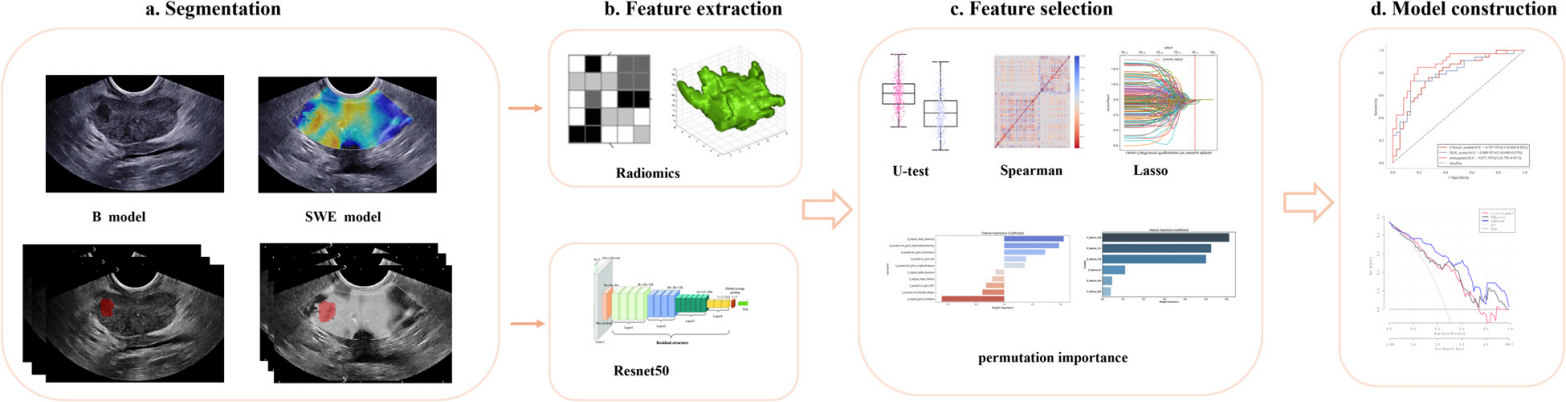
Developing a deep learning radiomic nomogram for predicting clinically significant prostate cancer involves four key stages: **(a)** ROI segmentation, **(b)** feature extraction, **(c)** feature selection, and **(d)** predictive modeling. Two predictive modeling strategies were used: the radiomics strategy involved feature extraction, reduction, and statistical modeling, while the deep learning strategy utilized ResNet-50, pretrained on ImageNet, for feature extraction. The final fully connected layer was removed, and global max pooling was applied to convert feature maps into raw values. Ultimately, the models developed through various strategies and utilizing different imaging modalities were integrated and evaluated for their applicability in clinical settings. ROI, region of interest.

For continuous variables, either the median with interquartile range (IQR) or the mean with standard deviation was used, and they were analyzed using the Mann–Whitney U test or the Kruskal–Wallis’ test, as needed. The sample size of this study adhered to the 10-events-per-variable (EPV) rule (15). Missing data, when less than 20%, were addressed using Multiple Imputation by Chained Equations (MICE). The AUC, along with 95% confidence intervals (CIs), was applied to quantify the capability of each model. The DeLong test was employed to assess the statistical significance of discrepancy in AUC values among the models. To enhance decision-making, a nomogram for the integrated model was developed. Additionally, decision curve analysis was employed to appraise the clinical utility of the clinical model, DLR score, and nomogram. Statistical significance was indicated by a two-tailed P-value below 0.05. R software (version 4.2) and Python (versions 3.7 and 3.9) were employed for the analyses outlined above. **Figure 3** illustrated the complete workflow of this analysis.

## 3 Results

### 3.1 Patients’ characteristics

Between June 2022 and December 2023, 272 patients with PSA rising and/or positive DRE were enrolled, with 232 meeting the inclusion criteria for this study. A detailed description of the baseline characteristics can be found in **Table 1**. There was no significant difference in the proportion of csPCa between the training and the test sets (56.17% [91/162] vs. 47.14% [33/70], *P* = 0.206). Significant differences between csPCa and non-csPCa groups, which included benign tissue and GS 3 + 3 PCa, were revealed by univariate analysis in both two sets for all clinical factors, except free prostate-specific antigen (fPSA). Subsequently, a clinical model was developed using multivariate analysis incorporating these factors, which served as a baseline for evaluating the nomogram proposed in this study (**Table 2**).

**Table 1:** Baseline characteristics of patients.

Variable	Training set (n=162)			Validation set (n=70)		
	Non-csPCa (n=71)	csPCa (n=91)	*P* value	Non-csPCa (n=37)	csPCa (n=33)	*P* value
Age Median (IQR)	71.00 (67.00 – 76.00)	74.00 (69.00 – 78.50)	0.016	68.00 (60.00 – 74.00)	72.00 (69.00 – 77.00)	0.046
tPSA Median (IQR)	8.06 (5.88 – 10.85)	10.70 (6.83 – 16.53)	0.002	8.45 (5.66 – 11.20)	13.82 (9.37 – 17.02)	0.001
fPSA Median (IQR)	1.32 (0.96 – 1.86)	1.24 (0.81 – 1.82)	0.520	1.17 (0.83 – 1.69)	1.38 (1.10 – 1.65)	0.269
f/tPSA Median (IQR)	0.17 (0.11 – 0.22)	0.11 (0.08 – 0.14)	<0.001	0.15 (0.11 – 0.23)	0.10 (0.08 – 0.14)	0.003
PSAD Median (IQR)	0.18 (0.13 – 0.28)	0.35 (0.21 – 0.56)	<0.001	0.17 (0.12 – 0.23)	0.34 (0.26 – 0.55)	<0.001
Volume Median (IQR)	44.27 (31.19 – 58.21)	32.38 (23.78 – 41.72)	<0.001	42.68 (34.54 – 70.17)	34.24 (26.52 – 47.79)	0.016
Gleason score, n (%)			<0.001			<0.001
3 + 3	7 (9.86)	0		6 (16.22)	0	
3 + 4	0	28 (30.77)		0	9 (27.27)	
4 + 3	0	21 (23.08)		0	9 (27.27)	
≥4 + 4	0	42 (46.15)		0	15 (45.46)	

csPCa, clinically significant prostate cancer; PSA, prostate-specific antigen; PSAD, PSA density.

**Table 2:** The results of multivariate logistic regression.

	B	Wald	OR with 95%CI	*P*
(Intercept)	-2.313	1.177	0.099 (0.001~6.209)	0.278
Age	0.08	7.952	1.083 (1.027~1.149)	0.005
tPSA	0.03	0.111	1.03 (0.86~1.224)	0.739
f/tPSA	-14.177	5.411	0 (0~0.026)	0.020
PSAD	-0.483	0.051	0.617 (0.011~57.362)	0.821
Volume	-0.04	3.866	0.96 (0.921~0.999)	0.049

### 3.2 Image signature analysis

The consistency of feature extraction was evaluated through intra-observer and inter-observer assessments using intraclass correlation coefficients (ICCs). The results indicated that feature extraction demonstrated high reproducibility, with both inter-observer and intra-observer ICCs exceeding 0.8.

From single parametric ultrasound image per patient, we extracted a total of 851 features, yielding 1,702 features across bi-parametric ultrasound images. Furthermore, we derived 512×2 deep learning features from the average pooling layer of the ResNet-50 architecture for each individual. Following the feature selection process, we determined 10 radiomic features and 6 deep learning features, which were subsequently integrated to form DLR signatures (**Supplementary Figure S1**). With the exception of E_wavelet_LHH_firstorder_Median, all features demonstrated statistically significant differences between the csPCa and non-csPCa groups (*P* < 0.05) (**Supplementary Figure S2**). The values of deep learning features were significantly higher in the csPCa group compared to the non-csPCa group (*P* < 0.001).

### 3.3 The development of deep learning radiomics model

**Figure 4:**
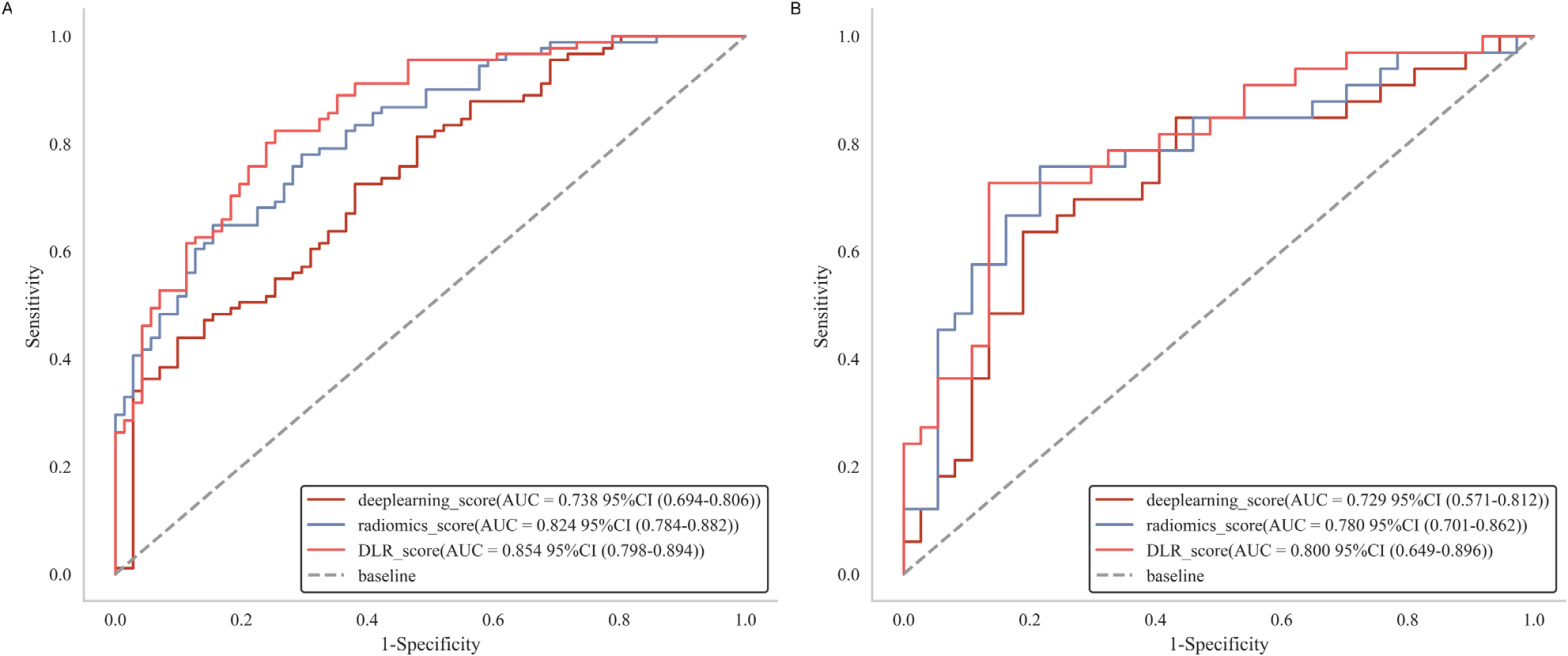
Receiver operating characteristic curves of radiomics score, deep learning score and DLR score, respectively in the training **(A)** and test **(B)** cohorts. DLR, deep learning radiomics.

Following the selection of features, we evaluated multiple modeling techniques to identify the most effective approach for model construction. Among these models, the LR model revealed superior diagnostic performance, achieving an AUC of 0.78 (95%CI: 0.61-0.94) on the test set. The diagnostic performance metrics for the remaining models are presented in **Supplementary Figure S3**. For each patient, we computed the radiomics score, deep learning score, and DLR score utilizing the weight coefficients derived from multivariate analysis, with the specific formulas detailed in Appendix 1. Our findings indicated that the DLR score achieved an AUC of 0.80 (95% CI: 0.69-0.91) in the test set, marginally surpassing the radiomics score (AUC = 0.78, 95% CI: 0.67-0.89) and outperforming the deep learning score (AUC = 0.73, 95% CI: 0.61-0.85), as illustrated in **Figure 4**. Furthermore, the specificity and accuracy of the DLR score were 0.87 (95% CI: 0.70-0.99) and 0.77 (95% CI: 0.54-0.91), respectively, surpassing those of the radiomics. However, there is no significant difference between DLR score and both radiomics (P = 0.48) and deep learning (P = 0.2) in the test set. Additional information is provided in **Supplementary Tables S1** and **S2**.

### 3.4 The development of the nomogram

**Figure 5:**
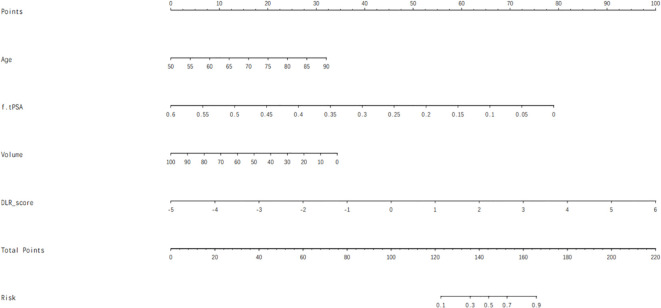
Nomogram that integrated DLR score and clinical factors for predicting csPCa.

In our study, multivariate analysis revealed that age, free-to-total prostate-specific antigen (f/t PSA) ratio, and prostate volume were independent predictors for csPCa within the training set, with statistical significance indicated by P-values less than 0.05 (**Table 2**). These independent predictors were subsequently combined with the DLR score to construct a composite model, which is visually represented as a nomogram (**Figure 5**).

### 3.5 The evaluation of the nomogram

**Figure 6:**
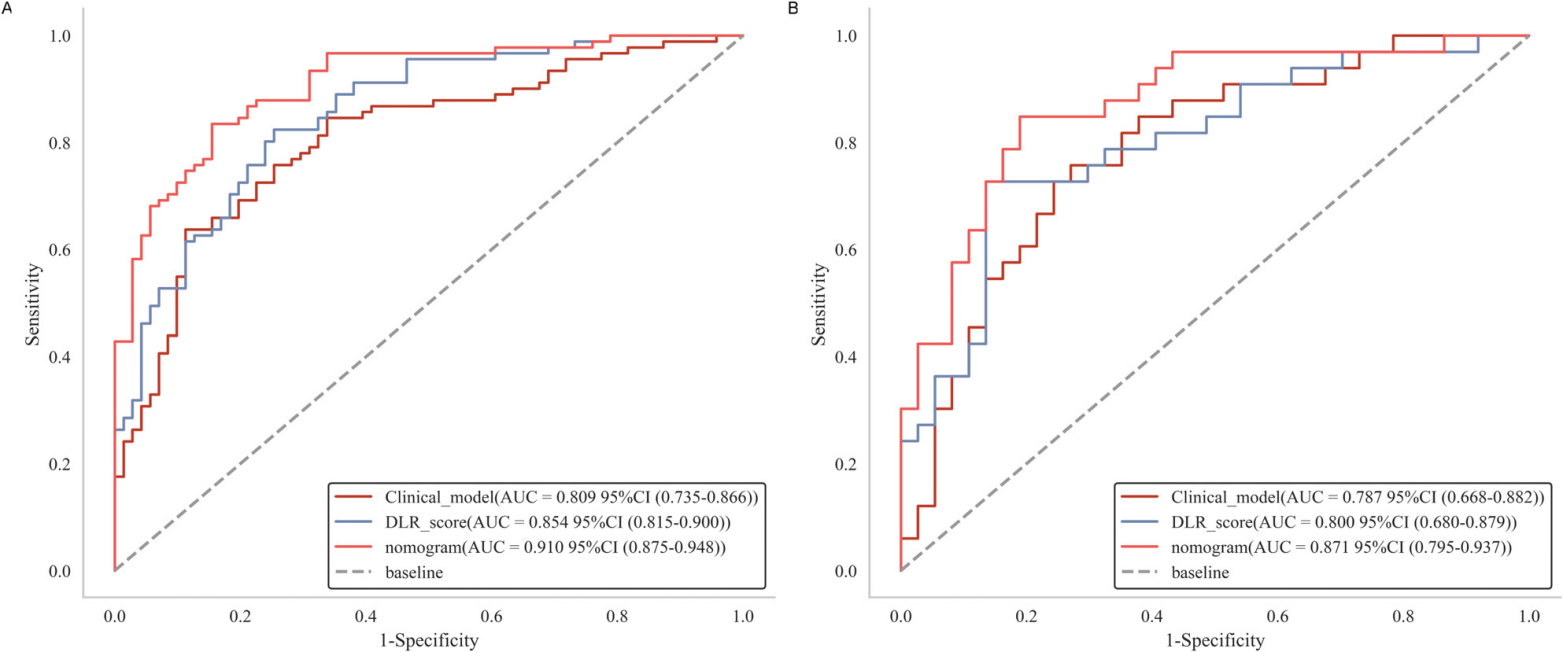
Receiver operating characteristic curves of clinical model, DLR score and nomogram in the training **(A)** and test **(B)** cohorts.

The nomogram demonstrated excellent diagnostic performance, achieving an AUC of 0.91 (95% CI: 0.87-0.95) in the training set and 0.87 (95% CI: 0.77-0.95) in the test set (**Figure 6**). Furthermore, the Delong test indicated a statistically significant difference between the nomogram and both the DLR score (P = 0.049) and the clinical model (P = 0.041) in the test set, underscoring the superior discriminative capability of the nomogram for detecting csPCa (**Table 3**). No significant difference was observed between the DLR score and the clinical model (**Supplementary Table S3**). Furthermore, the performance of the nomogram was assessed across different threshold levels in the test set, as detailed in **Supplementary Table S4**. A threshold value of 0.59 was identified as optimal, demonstrating a balance between sensitivity and specificity, with 0.82 and 0.84, respectively. A threshold of 0.19 exhibited a high sensitivity of 0.94, while a threshold of 0.89 achieved a high specificity of 1.0.

**Table 3:** Diagnostic performance of the models.

	Clinical model		DLR score		Nomogram	
	Training	Test	Training	Test	Training	Test
AUC (95%CI)	0.81 (0.71-0.87)	0.79 (0.69-0.86)	0.85 (0.79-0.91)	0.80 (0.70-0.91)	0.91 (0.87-0.95)	0.87 (0.77-0.95)
Sensitivity (95%CI)	0.64 (0.57-0.87)	0.76 (0.60-0.97)	0.82 (0.69-0.96)	0.73 (0.59-0.91)	0.84 (0.72-0.98)	0.85 (0.69-1.00)
Specificity (95%CI)	0.89 (0.58-0.95)	0.73 (0.52-0.95)	0.75 (0.57-0.91)	0.87 (0.76-0.97)	0.85 (0.68-0.94)	0.81 (0.65-0.94)
Accuracy (95%CI)	0.77 (0.69-0.82)	0.77 (0.68-0.84)	0.80 (0.74-0.86)	0.78 (0.51-0.91)	0.85 (0.80-0.90)	0.84 (0.75-0.91)
Youden index (95%CI)	0.53 (0.40-0.65)	0.49 (0.37-0.66)	0.57 (0.46-0.72)	0.59 (0.43-0.81)	0.68 (0.60-0.80)	0.66 (0.49-0.83)

DLR, deep learning radiomics; AUC, area under curve.

**Figure 7:**
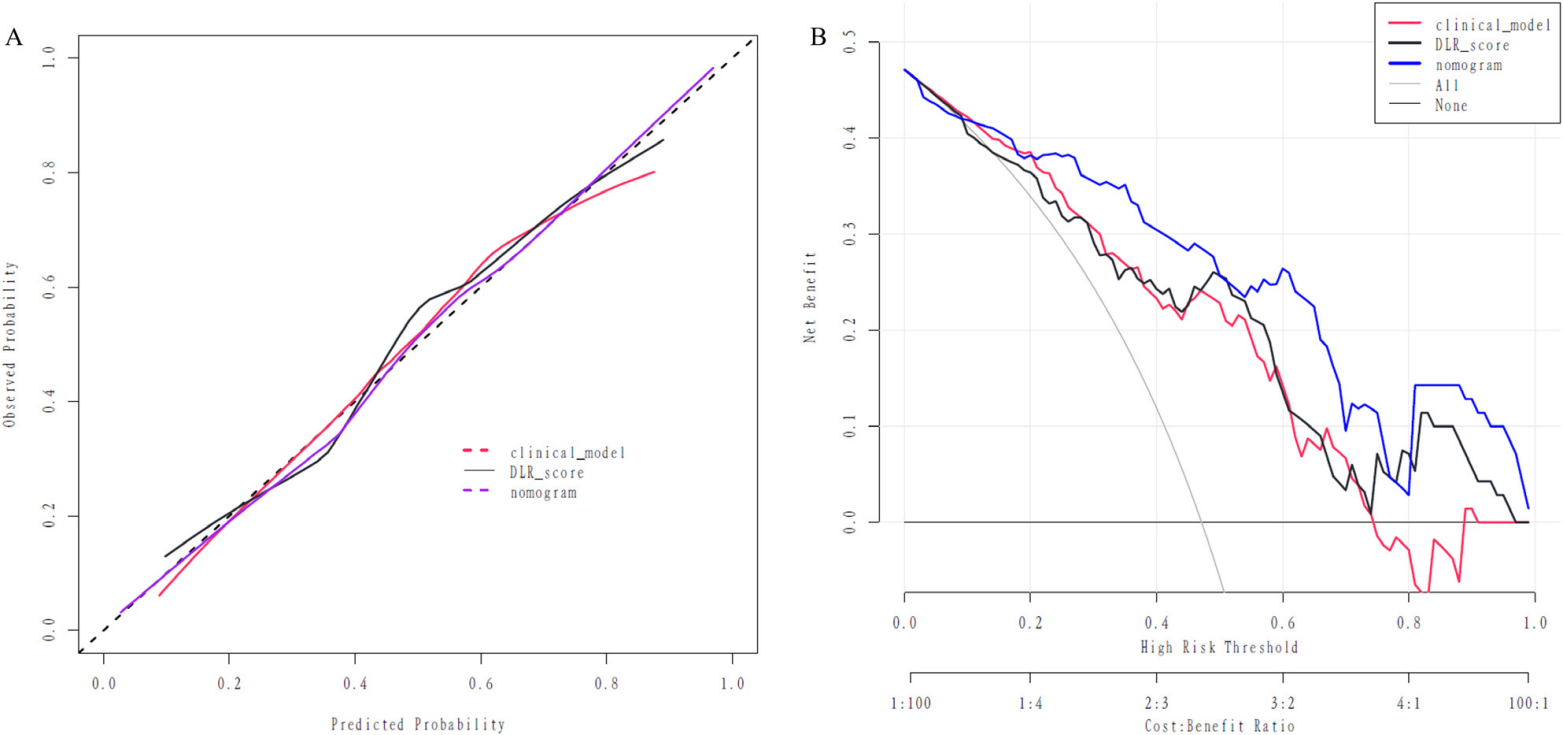
The assessment of the nomogram via calibration curve **(A)** and Decision curve **(B)**.

Our study’s calibration curve showed strong agreement between the nomogram’s predicted probabilities and the actual outcomes (**Figure 7A**). Furthermore, the results of our decision curve analysis, depicted in **Figure 7B**, illustrated that the nomogram provided substantial clinical decision-making benefits, with an effective threshold range of ≥15% in the test set.

## 4 Discussion

The study focused on developing and validating a mixed model presented by nomogram, which integrated a bi-parametric ultrasound-based DLR with clinical factors. This integration enhanced the accuracy of early identification of csPCa in patients with suspected PCa prior to undergoing prostate biopsy. Compared to the clinical model and the DLR score separately, the nomogram exhibited enhanced diagnostic performance, achieving an AUC of 0.87 (95% CI: 0.77-0.95) in the test set. Furthermore, the results from calibration and decision curve analyses corroborated the model’s robustness and clinical applicability. To our understanding, this study was the first to merge ultrasound-based radiomics with deep learning features to assess the risk of csPCa in individuals suspected of PCa.

To date, multiparametric MRI (mpMRI) has been recommended by guidelines for further identification of PCa lesions, thereby reducing the need for unnecessary biopsies (16). Several studies have been carried out to enhance the accuracy of csPCa detection using the Prostate Imaging—Reporting and Data System (PIRADS) score, both showing improved diagnostic performance (17, 18). However, the limited availability of mpMRI, along with contraindications or intolerance to MRI in some patients, may restrict its widespread use. Consequently, there has been increasing interest among physicians in utilizing ultrasound as an alternative diagnostic tool for PCa detection due to their lower cost, wider accessibility, and easier integration into routine clinical workflows. Previous studies have demonstrated that multiparametric ultrasound detected 4.3% fewer csPCa cases compared to mpMRI, but resulted in 11.1% more patients being referred for biopsy (6). Considering its inferior diagnostic performance compared to MRI, further efforts are necessary to enhance its accuracy.

Artificial intelligence has profoundly altered medical practice by facilitating more rapid and precise data analysis, thereby revolutionizing diagnostic and therapeutic approaches (19). This technological advancement has necessitated a reevaluation of conventional screening methodologies. Deep learning techniques employed multi-layer convolution and filtering to generate ultra-high-dimensional features associated with diseases. Although these features are frequently challenging to interpret in a clinical context, they exhibited strong correlations with patient group classifications, leading to models with substantial clinical applicability (20). In our study, we deliberately eschewed end-to-end methodologies (21), instead prioritizing the integration of clinical scores to augment the interpretability of the model. As illustrated in **Supplementary Figure S2**, the deep learning features exhibited elevated values in the csPCa group compared to the non-csPCa group, potentially enhancing medical professionals’ comprehension of the predictive outcomes. Nevertheless, our methodology may result in an underutilization of intricate data patterns, thereby diminishing the model’s capacity to identify potential predictive signals and consequently affecting its accuracy. Further research is warranted to investigate strategies for enhancing the model’s accuracy while preserving its practicality and simplicity in clinical settings.

Radiomics and deep learning techniques have predominantly been applied in conjunction with mpMRI for the diagnosis of PCa (7, 22, 23), the grading of pathological features (10), and the prediction of biochemical recurrence (8). The diagnostic performance ranged from 0.788 to 0.958. Nevertheless, there is a notable absence of studies utilizing ultrasound-based deep learning approaches, with only a limited number of investigations focusing on ultrasound-based radiomics of prostate lesions. Liang et al. developed a radiomics model utilizing gray-scale ultrasound and SWE, and subsequently constructed an LR model by incorporating clinical factors and the radiomics score. This model reached an AUC of 0.90 for detecting PCa in the test set (13). Similarly, Sun et al. conducted a study involving 166 patients, demonstrating that a multiparametric ultrasound approach, which combined grey scale ultrasound with CEUS, obtained an AUC value of 0.89 for detecting peripheral zone PCa (24). In a study by Wildeboer et al, an automatically multiparametric ultrasound classifier was developed and demonstrated comparable diagnostic performance, with AUC of 0.75 and 0.90 for PCa and csPCa, respectively (25). These research underscores the potential role of ultrasound-based radiomics in PCa diagnosis. However, relying solely on the extraction of radiomic features may not capture the deeper, intrinsic characteristics of the tumor, thereby potentially limiting its broader applicability. In this research, we concurrently utilized radiomics and deep learning methodologies to develop a DLR model aimed at providing enhanced image information. Our findings demonstrated that the DLR score exhibited superior diagnostic performance in identifying csPCa compared to other models, including the radiomics score and deep learning score, achieving an AUC of 0.80 (95% CI: 0.68-0.91) in the test set. Nevertheless, no significant differences were perceived among the three models on the test set (**Supplementary Table S2**), indicating only marginal improvement when deep learning features were integrated with radiomic features. This outcome may be attributed to the delineation of prostate lesion by employing manual segmentation method in this study, which is specifically tailored for radiomics. Li et al. reported that a rectangular bounding box was employed to delineate the whole prostate instead of the segmentation of the prostate lesions for deep learning purposes (7). We hypothesized that segmentation for deep learning requires a broader scope to facilitate the extraction of more comprehensive information. There is a need for more research and exploration in this subject.

Over the past few decades, numerous studies have been conducted on the prediction of PCa; however, an optimal model based on prostate-related clinical factors has yet to be established. Utilizing multivariate analyses, we identified age, f/tPSA, and prostate volume as significant clinical risk factors. Chen et al. demonstrated that age, positive DRE, f/tPSA, and PSA density were independent clinical risk factors of PCa, achieving an AUC of 0.82 (26), similar to our study’s results. Wang et al. conducted a systematic review of ten studies and reported that the use of the f/tPSA maintained high diagnostic accuracy, with a summary ROC of 87% (27). Nonetheless, several studies have identified PSA density as an independent risk factor rather than the f/tPSA. Differences in clinical factors selected in each study and inconsistent case grouping may account for this discrepancy. Furthermore, our study identified prostate volume as an independent predictor of csPCa, aligning with findings from previous research. According to Porcaro et al., a larger prostate volume index was connected to a lower risk of high tumor burden and was related to reduced biological aggressiveness of prostate cancer in patients who underwent initial random biopsies (28).

Furthermore, there are a few limitations to this study. First, the retrospective design of this single-center study, which included only 232 patients for the development of the DLR model, potentially diminished the statistical power and may constrain the generalizability of the findings. This issue is recurrent in the field of radiomics research, as evidenced by other studies with smaller sample sizes, encompassing 166 (24), 112 (13), and 103 (29) patients. The results of our study are promising, as they furnish preliminary evidence supporting the correlation between multi-parametric ultrasound-based deep learning radiomics and patients with csPCa, thereby presenting a viable alternative for those unable to undergo MRI examinations. Moreover, prior research has demonstrated that model performance can deteriorate due to inconsistencies in data collection protocols, patient heterogeneity, and the challenges of external data validation (30). Consequently, our findings necessitate a larger sample size and external validation prior to their application in clinical practice. Future research will focus on expanding the patient sample size by prolonging the enrollment period and may potentially develop this study into a multi-center research project. Secondly, in our study, manual segmentation was utilized to define the region of interest (ROI), which may result in reduced reproducibility, poor inter-operator consistency, and a process that was both time-consuming and labor-intensive. Although existing literature has explored automated segmentation methods for delineating the prostate gland (23, 31), the automatic segmentation of lesions remains challenging. Given the limitations associated with the low detection rate of prostate lesions using grayscale ultrasound and the registration of multi-parametric ultrasound images in our study, automatic segmentation for delineating the ROI may not necessarily be less effective than manual segmentation. Consequently, we propose the future implementation of deep learning techniques to automatically delineate the entire prostate as the ROI and to employ deep learning-based visual analysis for the identification of suspicious lesions. This approach is anticipated to enhance reproducibility and reduce the labor burden, thereby facilitating large-scale studies in the future. Thirdly, while the DLR score demonstrated superior diagnostic efficacy in our study, it was not directly compared with the PIRADS score, a widely utilized scoring system based on mpMRI in clinical practice. Future research should incorporate PIRADS scores to further validate the model’s effectiveness.

In our study, the nomogram developed by integrating DLR score with clinical factors, demonstrated high diagnostic performance and clinical utility in identifying csPCa. By combining deep learning with radiomics, the model could effectively capture the multidimensional information inherent in imaging data, thereby enhancing radiologists’ confidence in predicting csPCa in future clinical practice.

## Data Availability

The data supporting the findings of this study can be accessed from the corresponding author upon a reasonable request. Requests to access these datasets should be directed to H-MH, ntyyhhm123@163.com.

## Appendix 1

Formulas of deep learning radiomics model.

Radiomics_score:

= 0.3488 + (-0.6361) * E_original_shape_Flatness + (0.3754) * E_original_ngtdm_Busyness + (-0.4165) * E_wavelet.LLH_glcm_MCC + (-0.3461) * E_wavelet.LHH_firstorder_Median + (0.4584) * E_wavelet.HLH_glrlm_LongRunEmphasis + (0.3724) * E_wavelet.LLL_glcm_Idm + (0.6147) * B_original_shape_Sphericity + (-0.9829) * B_original_glcm_Correlation + (0.8695) * B_wavelet.LHH_glszm_SizeZoneNonUniformity + (0.7131) * B_wavelet.HLH_glrlm_RunVariance

Deep learning_score:

=0.3480 + (0.2547) * E_feature_91 + (0.3263) * E_feature_168 + (-0.0296) * E_feature_264 + (0.2842) * B_feature_111 + (0.3121) * B_feature_159 + (0.2215) * B_feature_424

DLR_score:

= 0.4176 + (-0.2110) * E_original_shape_Flatness + (-0.1514) * E_original_ngtdm_Busyness + (-0.3182) * E_wavelet.LLH_glcm_MCC + (-0.3849) * E_wavelet.LHH_firstorder_Median + (0.3617) * E_wavelet.HLH_glrlm_LongRunEmphasis + (0.3862) * E_wavelet.LLL_glcm_Idm + (1.0507) * B_original_shape_Sphericity + (-1.1021) * B_original_glcm_Correlation + (0.9667) * B_wavelet.LHH_glszm_SizeZoneNonUniformity + (0.7215) * B_wavelet.HLH_glrlm_RunVariance + (0.1090) * E_feature_91 + (0.6123) * E_feature_168 + (0.0398) * E_feature_264 + (0.5253) * B_feature_111 + (0.5) * B_feature_159 + (0.046) * B_feature_424
