# Supplementary material for: A retrospective study on predicting clinically significant prostate cancer via a bi-parametric ultrasound-based deep learning radiomics model
Source: Front Oncol. 2025 Apr 8;15:1538854. doi: 10.3389/fonc.2025.1538854 (PMC12011619; doi:10.3389/fonc.2025.1538854)
Supplement: Supplementary file 1 [file DataSheet1.docx]

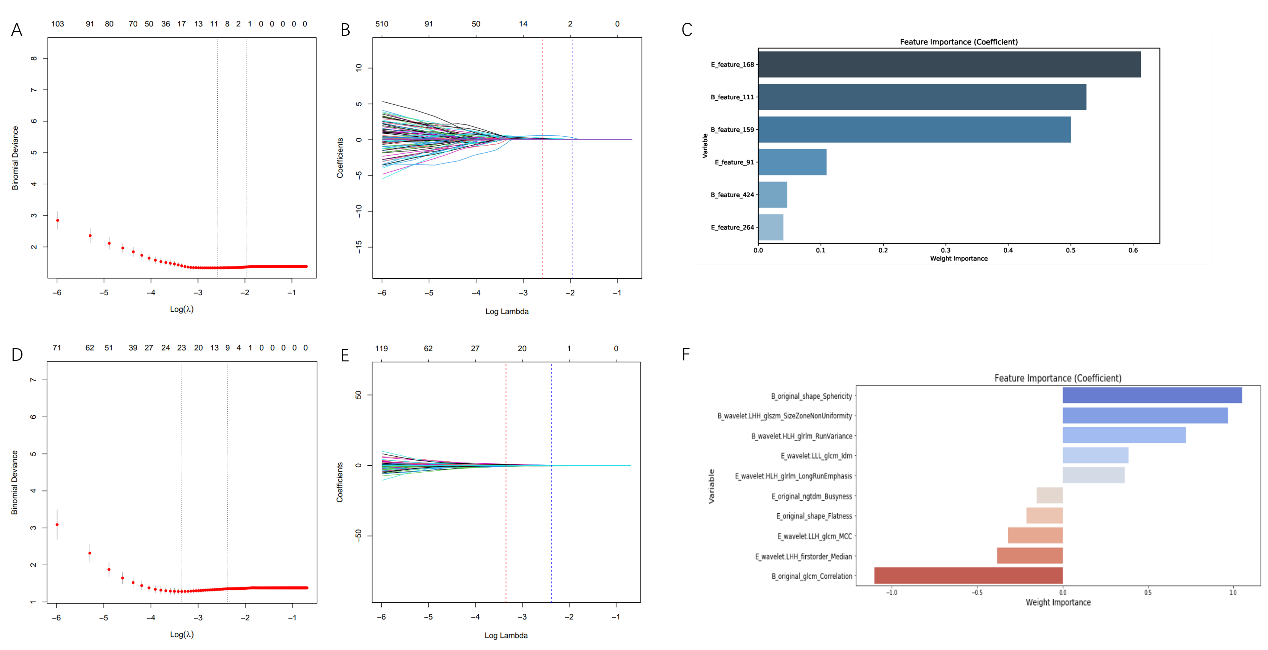


Figure S1: further selecting deep learning features (A-C) and radiomics features (D-F) via the LASSO method and permutation importance method; (A) and (D) The determination of the key parameter (penalty coefficient: l) in the LASSO model using 10-fold cross-validation. Two rules resulted in two l values (λmin: when the prediction error reached the minimum and λ1se: the value within one standard error from the minimum) and two vertical dashed lines at their position were drawn. λmin was adopted in the feature selection of LASSO in this study; (B) and (F) Feature coefficients profiles as the λ value changes. According to the 10-fold cross-validation in (A) and (D); Importance and ranking of features associated with clinical significantly prostate caner diagnosis using random forest modelling. 6 features were finally selected with best AUC in the deep learning features (C), and 10 features were finally identified in the radiomics features (F).


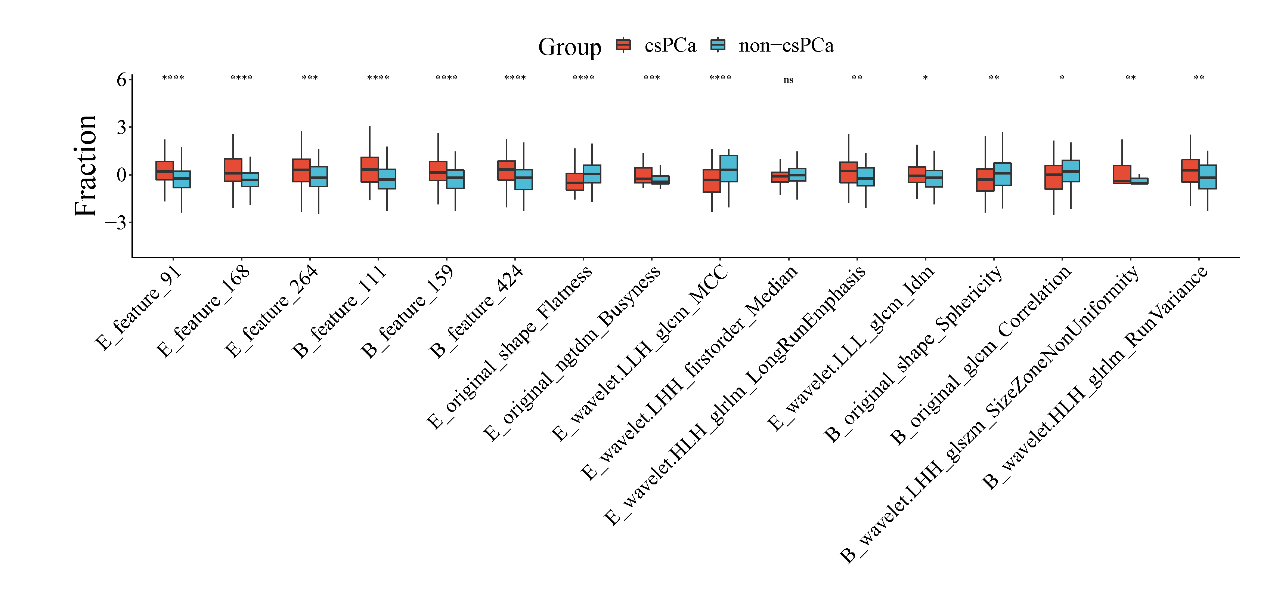


FigureS2. Box plots represent the differences in signatures between the csPCa and non-csPCa groups. This box plot illustrates the distribution of data using the mean and quartiles.


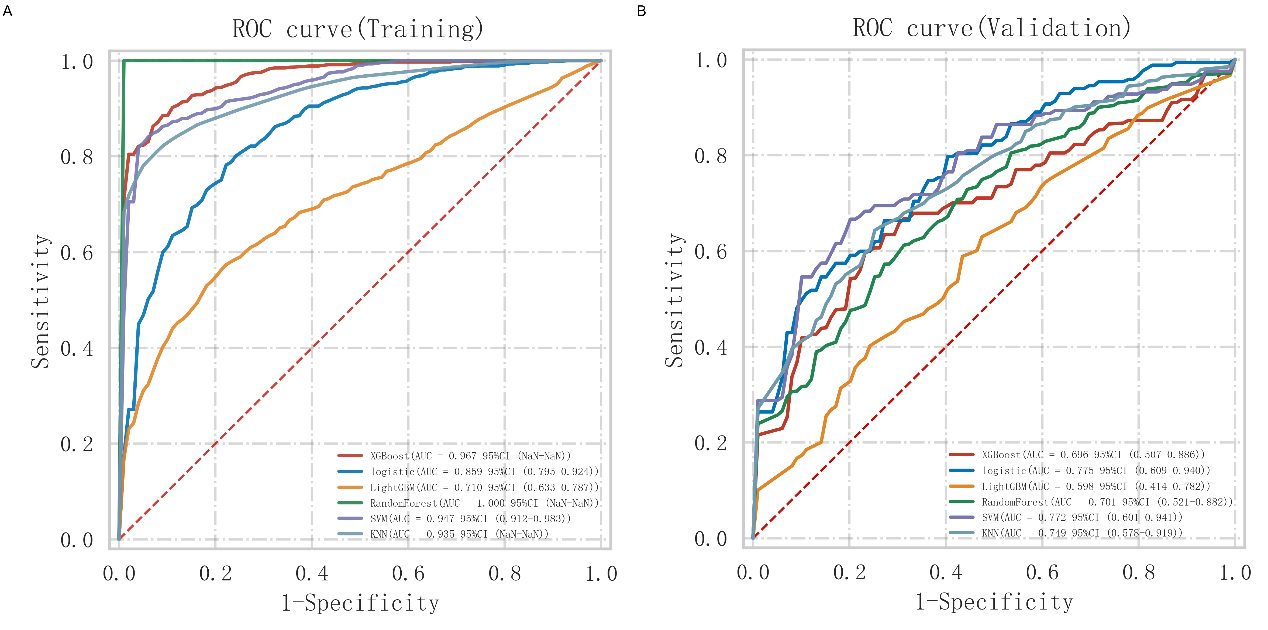


Figure S3. Receiver operating characteristic curves among different machine learning methods of deep learning radiomics signatures in the training (A) and test (B) cohorts.

Table S1: AUC results of deep learning score, radiomics score, and DLR socre for predicting csPCa

|  | Deep learning score | | Radiomics score | | DLR score | |
| --- | --- | --- | --- | --- | --- | --- |
|  | training | test | training | test | training | test |
| AUC  (95%CI) | 0.74  (0.65-0.80) | 0.73  (0.60-0.82) | 0.82  (0.77-0.90) | 0.78  (0.69-0.88) | 0.85  (0.79-0.91) | 0.80  (0.68-0.91) |
| Sensitivity (95%CI) | 0.73  (0.38-0.90) | 0.64  (0.51-0.91) | 0.65  (0.52-0.91) | 0.76  (0.52-0.91) | 0.82  (0.71-0.96) | 0.73  (0.62-0.85) |
| Specificity (95%CI) | 0.62  (0.49-0.98) | 0.81  (0.49-0.92) | 0.85  (0.61-0.96) | 0.78(0.68-0.97) | 0.75  (0.59-0.90) | 0.87  (0.70-0.99) |
| Accuracy (95%CI) | 0.67  (0.42-0.75) | 0.72  (0.56-0.81) | 0.72  (0.39-0.83) | 0.76  (0.49-0.84) | 0.80  (0.74-0.85) | 0.77  (0.54-0.91) |
| Youden index (95%CI) | 0.35  (0.31-0.50) | 0.45  (0.31-0.64) | 0.49  (0.42-0.64) | 0.54  (0.37-0.70) | 0.57  (0.48-0.69) | 0.59  (0.43-0.81) |

Abbreviation: DLR, deep learning radiomics; 95%CI: 95% confidence interval

Table S2：Delong test results between different deep learning radiomics models in the test set.

|  | Deep learning score | Radiomics score | DLR score |
| --- | --- | --- | --- |
| Deep learning score |  | 0.441 | 0.2 |
| Radiomics score | 0.441 |  | 0.478 |
| DLR score | 0.2 | 0.478 |  |

Table S3: Delong test results between different constructed models in the test set.

|  | Clinical model | DLR score | Nomogram |
| --- | --- | --- | --- |
| Clinical model |  | 0.861 | 0.041 |
| DLR score | 0.861 |  | 0.049 |
| Nomogram | 0.041 | 0.049 |  |

Table S4: nomogram’s performance metrics at various threshold levels in the test set

| Threshold | Sensitivity (95%CI) | Specificity  (95%CI) | PPV  (95%CI) | NPV  (95%CI) |
| --- | --- | --- | --- | --- |
| 0.19 | 0.94 (0.8-0.98) | 0.49 (0.33-0.64) | 0.62 (0.48-0.74) | 0.9 (0.7-0.97) |
| 0.59 | 0.85 (0.69-1.0) | 0.81 (0.65-0.94) | 0.82 (0.65-0.91) | 0.84 (0.69-0.92) |
| 0.89 | 0.3 (0.17-0.47) | 1.0 (0.91-1.0) | 1.0 (0.72-1.0) | 0.62 (0.49-0.73) |

Abbreviation: PPV, positive predictive value; NPV, negative predictive value
